# Pre-notifications increase retention in a 17-year follow-up of adolescents born very preterm

**DOI:** 10.1186/s13063-023-07390-1

**Published:** 2023-07-26

**Authors:** Minttu Helin, Max Karukivi, Päivi Rautava, Milka Hirvonen, Mira Huhtala, Sirkku Setänen, on behalf of the PIPARI Study Group, Mikael Ekblad, Satu Ekblad, Eeva Ekholm, Linda Grönroos, Leena Haataja, Laura Haveri, Eveliina Joensuu, Pentti Kero, Riikka Korja, Katri Lahti, Helena Lapinleimu, Liisa Lehtonen, Tuomo Lehtonen, Marika Leppänen, Annika Lind, Jonna Maunu, Petriina Munck, Eeva Mäkilä, Anna Nyman, Riitta Parkkola, Katriina Saarinen, Tiina Saarinen, Susanna Salomäki, Virva Saunavaara, Matti Sillanpää, Suvi Stolt, Karoliina Uusitalo, Milla Ylijoki

**Affiliations:** 1 Department of Pediatric Neurology, Turku University Hospital and the University of Turku, Savitehtaankatu 5, 20521 Turku, Finland; 2 Department of Adolescent Psychiatry, Turku University Hospital and the University of Turku, Kunnallissairaalantie 20, 20700 Turku, Finland; 3 Turku Clinical Research Center, Turku University Hospital, poBOX 52, 20521 Turku, Finland; 4 Public Health, University of Turku, 20014 Turun yliopisto, Turku, Finland; 5 The Faculty of Medicine, the University of Turku, Kiinamyllynkatu 10, 20520 Turku, Finland; 6 Department of Paediatrics and Adolescent Medicine, Turku University Hospital and the University of Turku, Savitehtaankatu 5, 20521 Turku, Finland

**Keywords:** Approval form, Long-term follow-up, Post-notification, Questionnaire, Response rate, Very preterm

## Abstract

**Objective:**

Retention is essential in follow-up studies to reduce missing data, which can cause bias and limit the generalizability of the results. We investigated whether pre-notification letters would increase the response rates of approval forms and questionnaires and reduce the need for post-notifications in a prospective follow-up study of 17-year-old adolescents.

**Study design:**

and settings

This long-term follow-up study included 269 adolescents were randomized (1:1) into a pre-notification group (*n* = 132) and a no pre-notification group (*n* = 137). The pre-notification letter was sent prior to the approval form and questionnaires. The outcome measures were the response rates to the approval forms and questionnaires and the rate of post-notifications required.

**Results:**

The adolescents who received the pre-notifications were more likely to return approval forms (*n* = 88/132, 67%) than the adolescents who did not receive the pre-notifications (*n* = 79/137, 58%) (OR 1.5, 95% CI 0.9–2.4). The rates of returned questionnaires were higher in the pre-notification group (*n* = 82/88, 93%) than in the no pre-notification group (*n* = 68/79, 86%) (OR 2.2, 95% CI 0.8–6.3). The adolescents who did not receive the pre-notifications were more likely to need the post-notifications than the adolescents who received the pre-notifications (OR 3.0, 95% CI 1.4 to 6.5).

**Conclusions:**

Pre-notifications decreased the need for post-notifications and may increase retention in 17-year-old adolescents. Based on our findings, pre-notification letters are recommended in future follow-up studies in adolescents.

**Trial registration:**

The Ethics Review Committee of the Hospital District of South-West Finland approved the 17-year PIPARI Study protocol in January 2018 (23.1.2018; 2/180/2012). The study has been registered to the SWAT repository as SWAT 179. Filetoupload,1457904,en.pdf (qub.ac.uk).

**Supplementary Information:**

The online version contains supplementary material available at 10.1186/s13063-023-07390-1.

## Introduction

Prospective follow-up studies provide valuable insight into the impact of a condition or treatment on patients’ lives. Participants staying in a study is called ‘retention’. Retention is essential to follow-up studies. The longer the follow-up time, the more difficult it is to maintain satisfactory retention. The reasons why participants discontinue participating in a study might be because they are busy, have difficulties coming to the clinic, or are just unwilling to contribute any longer. Low retention leads to missing data, which can cause a bias and limit the generalizability, validity, and reliability of the results. It has been considered that < 5% loss of participants is not problematic, but a loss of > 20% is a serious threat to the validity of the study [1, 2]. Walters et al. showed in their meta-analysis that the median loss-to-follow-up in a sample of 151 trials was 11% [3]. In most studies included in the meta-analysis, the follow-up time ranged from ≤ 18 months to up to 10 years. Studies within a trial (SWATs) are carried out within larger clinical trials to evaluate alternative strategies to improve the efficiency of the trial process. Treweek et al. have defined SWAT as “a self-contained study that has been embedded within a host trial with the aim of evaluating or exploring alternative ways of delivering or organizing a particular trial process” [4].

A recent Cochrane review identified 70 studies that evaluated interventions to improve trial retention [5]. Researchers have investigated many kinds of methods for retaining participants in studies and prior SWATs have investigated whether the following approaches could increase retention in adults: reminders (letter, card, post-it note, short message service, e-mail, telephone call), additional items (logo-sticker, pen, fridge magnet), personalized reminders, newsletters, theory informed letter, monetary incentives, a personalized photo on a letter, or the color of the envelope [5–12]. The Cochrane review reported that there was no study with high-certainty evidence as determined by the Grading of Recommendations, Assessment, Development and Evaluations (GRADE) assessment which is a structured framework for the systematic reporting of studies [13]. The literature on SWAT or embedded studies using postal or electronical pre-notifications is scarce, and there is no clear evidence about their retention effectiveness. As far as we know, there are no previous SWATs or embedded studies conducted on long-term follow-up studies including children or adolescents. Nevertheless, methods used in trials are expected to be appropriate also within prospective study design.

This study aimed to investigate whether sending a postal pre-notification would increase the return rate of approval forms or questionnaires and reduce the number of post-notifications needed in 17-year-old adolescents. We hypothesized that the pre-notifications would increase both response rates (approval forms and questionnaires) and reduce the need for post-notifications.

## Material and methods

### Study protocol

The study has been registered to the SWAT repository as SWAT 179. Filetoupload,1457904,en.pdf (qub.ac.uk)

### Trial design

**Fig. 1:**
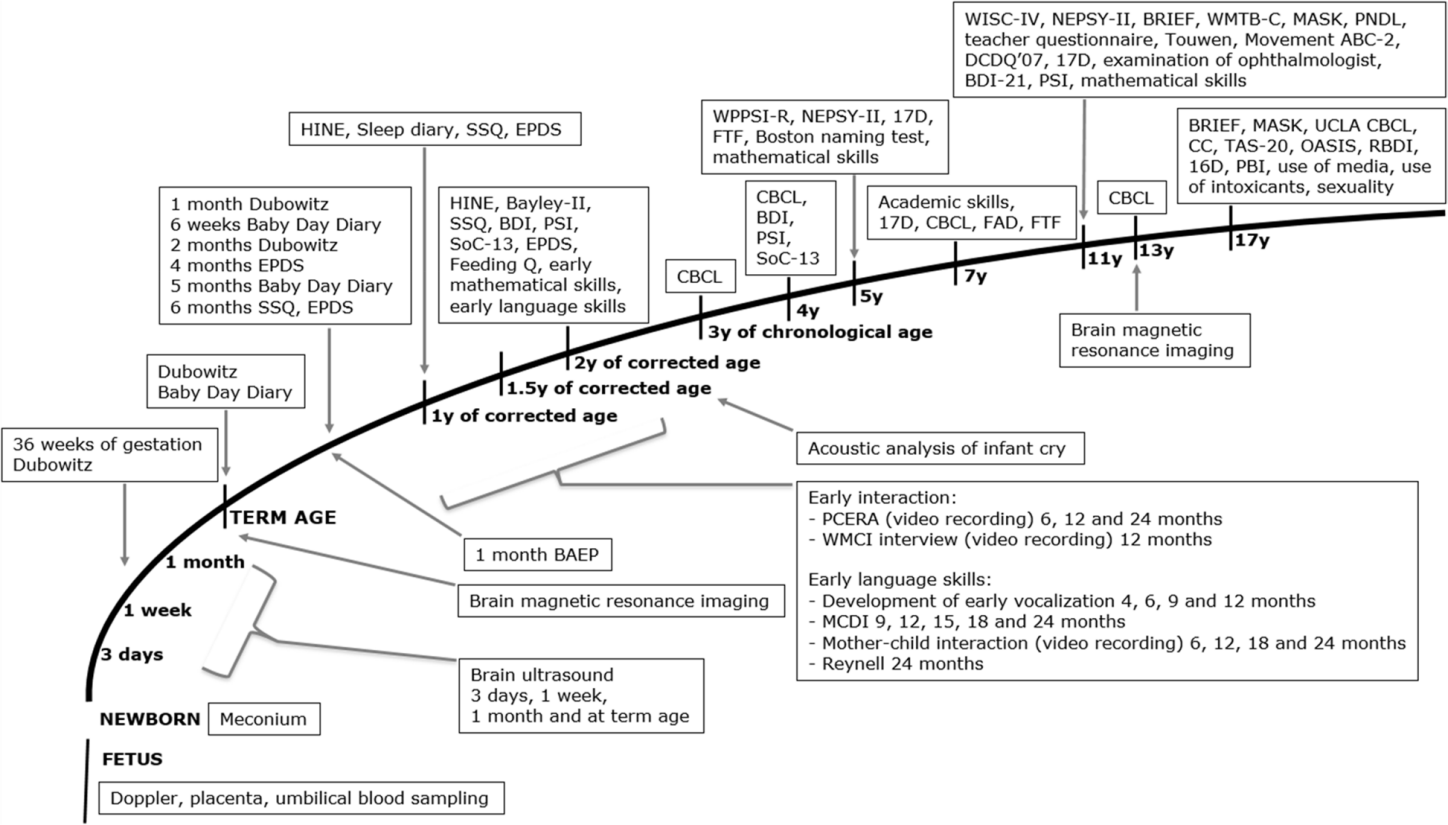
The study protocol of the host PIPARI Study of very preterm infants. Abbreviations with references are in Additional file 1. At the 17-year age-point (between January 2019 and December 2021), all the adolescents born between 2002 and 2004 and their parents who participated in the host PIPARI Study were included in this study. They were randomized into two groups (1:1): a pre-notification (*n* = 132 adolescents) and a no pre-notification (*n* = 137 adolescents). In the pre-notification group, adolescents were sent a pre-notification letter before the written information, the approval form, and the follow-up questionnaires. The Ethics Review Committee of the Hospital District of South-West Finland approved the 17-year PIPARI Study protocol in January 2018 (23.1.2018; 2/180/2012)

This study was part of the Finnish prospective multidisciplinary PIPARI Study (The Development and Functioning of very low weight infants from Infancy to School Age) [14]. The study protocol of the host PIPARI Study is described in detail in Fig. 1.

### Participants

**Fig. 2:**
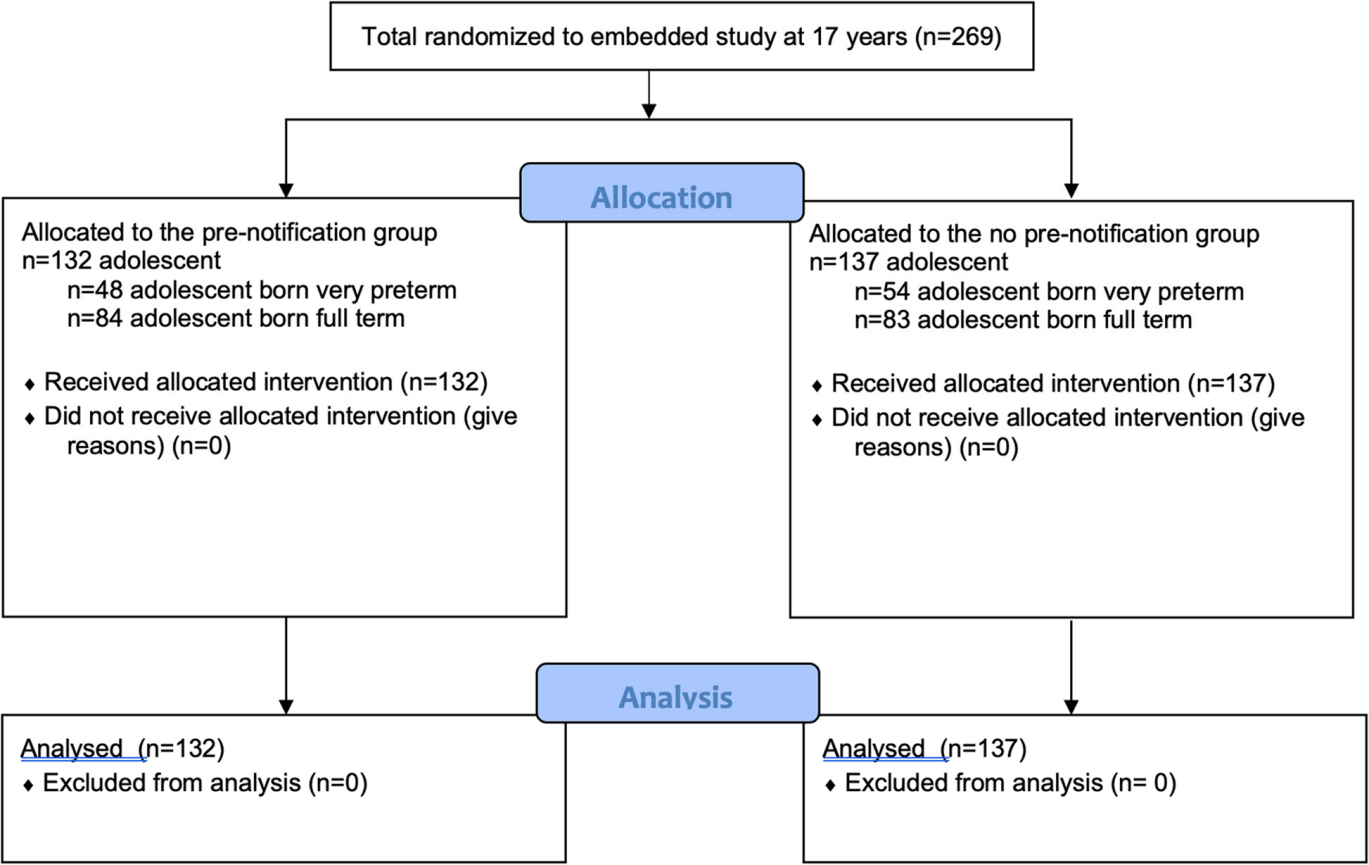
Flowchart of the study participants

The participants were born to Finnish- or Swedish-speaking families in Turku University Hospital, Finland, between 2002 and 2004. The inclusion criteria were birth weight ≤1500 g and gestational age < 37 weeks. From the beginning of 2004, the inclusion criteria were expanded to include all infants born < 32 gestational weeks, despite the birth weight. The exclusion criteria were severe congenital anomalies or a diagnosed syndrome affecting cognitive development. The control group consisted of healthy full term (> 37 weeks) infants born at Turku University Hospital during the same period. Families were informed about the host PIPARI Study protocol in the neonatal intensive care unit (very preterm infants) or at the newborn nursery (full term controls). At the 17-year age-point, all the adolescents born between 2002 and 2004, and their parents were included in this study. The flowchart of the participants is shown in Fig. 2.

### Intervention

**Fig. 3:**
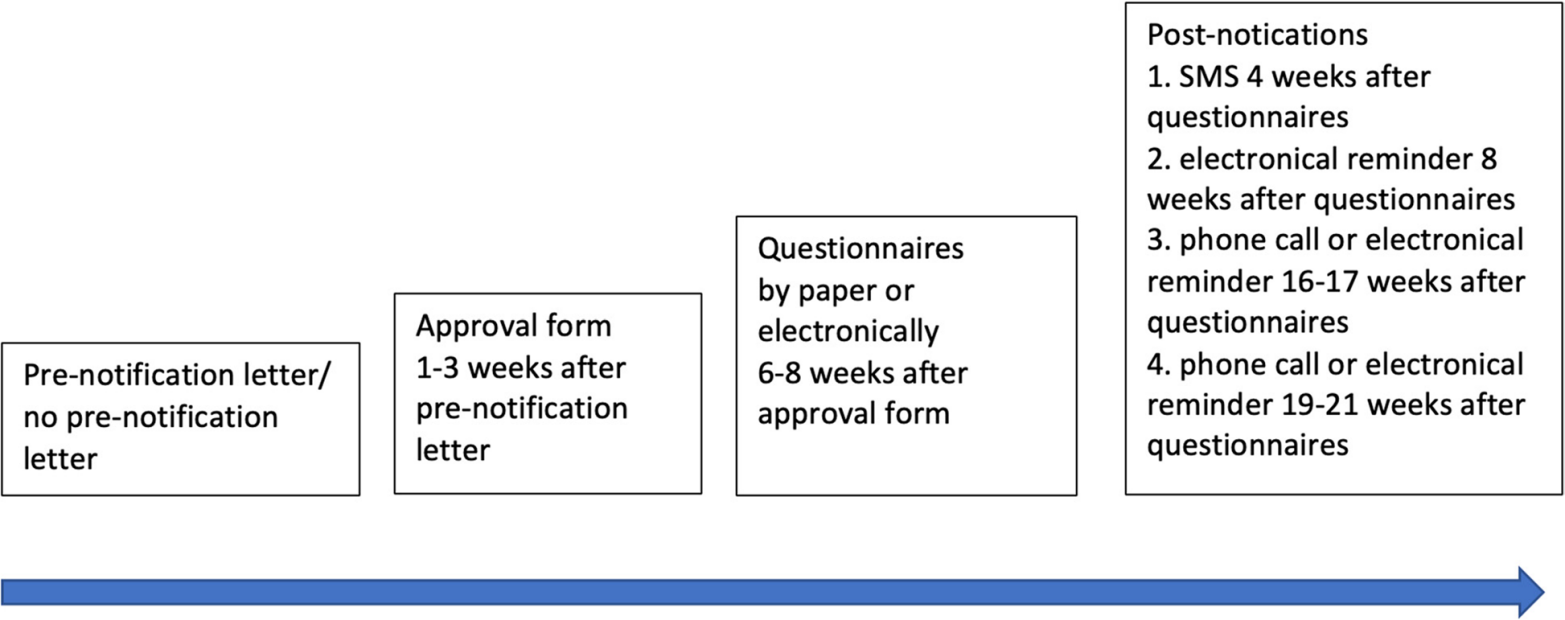
The flowchart of the Study protocol

The intervention aimed to discover whether the pre-notification letters increased the rate of the returned approval forms and questionnaires. The questionnaires related to mental health, behavior, quality of life, language skills, executive functions, substance abuse, use of media, sexuality, and parenthood. Adolescents randomized into the pre-notification group received the pre-notification letter (Additional file 2) 1–3 weeks prior to the study approval form, which was sent at the earliest 6–8 weeks before the study questionnaires, depending on the return of the approval form (Fig. 3). In the approval form, adolescents chose to complete and return the questionnaires by paper (*n* = 77) or electronically (*n* = 90) using the REDCap meta-data driven software toolset [15]. If the adolescent had not returned the questionnaires within 4 weeks, the first post-notification, a short message service (SMS) was sent by the study coordinator. In cases where the forms had not been returned after 8 weeks, a second post-notification phone call was made or an electronical reminder was sent. When necessary, third and fourth post-notifications were made by telephone or by an electronical reminder. This study followed the CONSORT 2010 guidelines, which is a protocol for reporting the results of randomized clinical trials [16].

### Outcomes

The primary outcome measure was the response rate of approval forms. The secondary outcomes were the rate of returned questionnaires and the rate of post-notifications (calls, SMSs, electronical reminders). The outcomes were assessed by including all the adolescents (both birth groups) in order to compare the pre-notification group and no pre-notification group.

### Sample size

A power calculation was not performed as embedded studies are not powered to detect a difference because of limited sample size by the host studies.

### Randomization

Every participant in the study has a unique identification number (ID). Participants were assigned to the group using the random permuted block randomization employed by the SAS software, Version 9.4, of the SAS System for Windows (SAS Institute Inc., Cary, NC, USA) with a block size of 12. Computer-generated randomization allocated the participants to either pre-notification or no pre-notification group. Gender and twins together with triplets were considered as stratification factors. Twins (*n* = 9) and triplets (*n* = 2) were randomized as one entity (pre-notification or no pre-notification group). The randomization was performed separately for adolescents born very preterm and full term controls. A statistician performed the randomization code. The statistician responsible for generating the allocation sequence and assigning the pre-notification and no pre-notification groups was not involved in the PIPARI Study.

### Blinding

The participants of the study were not aware of the study intervention (pre-notifications). The study coordinator managed the pre- and the post-notifications after randomization without blinding. There was no blinding either of the rest of the study team members.

### Statistical methods

The normality of the distributions was assessed both graphically and with the Shapiro-Wilk test. The normally distributed variables were described by means (SD). Continuous variables were compared between the adolescents and drop-outs in the study using the independent sample *t*-test. Comparisons between two categorical variables were done using the Pearson chi square or Fisher’s exact test, as appropriate. ORs and 95% CIs were computed using logistic regression to assess the impact of intervention. All analyses were conducted unadjusted and adjusted. The regression analyses were adjusted with parents’ educational level as it was the only statistically significant covariant. Also, socioeconomic status has been suggested to predict discontinuation [17, 18]. The analyses were not adjusted for stratification factors. Statistical analyses were performed using SPSS version 28. A two-sided *p*-value < 0.05 was considered statistically significant.

## Results

A total of 269 adolescents were included and randomized to receive or not to receive the pre-notification letter before receiving the approval form and the questionnaires (Fig. 2). Of the 132 adolescents (49.1%) in the pre-notification group, 48 (36.4%) were born very preterm and 84 (63.6%) full term. A total of 137 (50.9%) adolescents were randomized in the no pre-notification group, out of which 54 (39.4%) adolescents were born very preterm and 83 (60.6%) full term. Within adolescents born very preterm, perinatal background characteristics were compared between the adolescents randomized in pre-notification group and in no pre-notification group to study the balance of the groups at baseline (Table 1). The comparison indicated no statistically significant differences between the groups. The equivalent information regarding controls born full term was not available. One adolescent had surrogate parents, one had a stepmother, and one had a stepfather, who all participated in the study.

**Table 1:** Background characteristics of the adolescents born very preterm (birth weight ≤ 1500 g or gestational age < 32 weeks). Continuous variables were compared using the independent sample *t*-test, and comparisons between two categorical variables were performed using the Pearson chi square

Adolescents born very preterm	Pre-notification group, *n* = 48	No pre-notification group, *n* = 54
Gestational age, mean (SD), week	28.4 (2.9)	29.1 (2.6)
Birth weight, mean (SD), grams	1023.8 (268.8)	1139.7 (307.7)
Birth weight *z*-score, mean (SD)	− 1.4 (1.5)	− 1.3 (1.6)
Small for gestational age (< − 2 SD), *n* (%)	14 (29.2)	16 (29.6)
Male, *n* (%)	24 (56.2)	27 (50.0)
Cesarean delivery, *n* (%)	27 (56.3)	36 (66.7)
Multiple birth, *n* (%)	14 (29.2)	18 (33.3)
Bronchopulmonary dysplasia, *n* (%)	6 (12.5)	9 (16.7)
Operated necrotizing enterocolitis, *n* (%)	2 (4.2)	2 (3.7)
Sepsis, *n* (%)	8 (16.7)	10 (18.5)
Laser-treated retinopathy of prematurity, *n* (%)	2 (4.2)	3 (5.6)
Major brain pathologies in magnetic resonance imaging at term age^a^, *n* (%)	10 (20.1)	14 (25.9)
Mother’s education > 12 years, *n* (%)	16 (33.3)	23 (42.6)
Father’s education > 12 years, *n* (%)	10 (20.1)	10 (18.5)

^a^Setänen et al. have published in 2013 the specific MRI protocol and details about the classification of the findings [19]

The rates of returned approval forms and questionnaires were higher in the pre-notification group than in the no pre-notification group as shown in Table 2. The adolescents (*n* = 132) who did receive the pre-notifications were more likely to return approval forms and questionnaires than the adolescents (*n* = 137) who did not receive the pre-notifications (OR 1.5, 95% CI 0.9–2.4, and OR 2.2, 95% CI 0.8–6.3). These differences were not statistically significant, not even when adjusted with the mothers’ (approval forms OR 1.5, 95% CI 0.5–4.4 and questionnaires OR 1.2, 95% CI 0.3–4.6) or fathers’ educational level (approval forms OR 2.9, 95% CI 0.3−30.4 and questionnaires OR 0.3, 95% CI 0.0−3.5). The adolescents who did not receive the pre-notifications were more likely to need the post-notifications than the adolescents who received the pre-notifications (OR 3.0, 95% CI 1.4 to 6.5) also when adjusted with the mothers’ (OR 2.3, 95% CI 1.0–5.3) or fathers’ educational level (OR 2.6, 95% CI 1.1–6.5). These differences regarding the need for post-notifications between the groups were statistically significant also when analyzed separately according to the birth group (very preterm and full-term controls); however, they were not significant when adjusted for the mothers’ or fathers’ educational level within the birth groups.

**Table 2:** The unadjusted results between the pre-notification and no pre-notification groups of adolescents. Comparisons between the groups were performed using the Pearson chi square. ORs and 95% CIs were computed using logistic regression

	Adolescents in the pre-notification group, % (*n*)	Adolescents in the no pre-notification group, % (*n*)	*p*-value	OR	95% CI
Approval forms returned	66.7 (88/132)	57.7 (79/137)	0.1	1.5	0.9–2.4
Questionnaires returned	93.2 (82/88)	86.1 (68/79)	0.1	2.2	0.8–6.3
Need for post-notifications					
No post-notifications	35.2 (31)	15.2 (12)	0.003	3.0	1.4–6.5
At least one post-notification	64.8 (57)	84.8 (67)			

## Discussion

This study provides novel information on the effect of pre-notification letters on retention and the need for post-notifications within a 17-year prospective follow-up study of adolescents born very preterm. As hypothesized, sending pre-notifications decreased the need for post-notifications and may increase retention.

It might be challenging to obtain participants’ postal or electronic addresses in prospective follow-up studies. Monetary incentive is suggested as a retention increasing method, but it is against Finnish research regulations [20]. Many different study protocols have evaluated methods to increase retention of the follow-up studies. The present study is the first embedded follow-up study including children or adolescents. In the SWAT repository, one registered ongoing pre-notification protocol (SWAT 86) investigated the effect of pre-notification letters on questionnaire response rates in adults. Previous SWATs have investigated the effect of the pre-notification SMS on the retention rate of the questionnaires in trials regarding adults [6, 7, 9, 21, 22]. In contrast to our findings from the follow-up study including adolescents born very preterm and full term, none of these trials reported a difference in response rates in adults. However, as there are no previous literature about improving retention in a follow-up study of adolescents, methods used in trials are expected to be appropriate also within prospective study design. The effect of electronic message timing on response rate has been investigated previously [23]. Post-notifications were found to be more effective than pre-notifications. Keding et al. found both pre- and post-notifications ineffective [21]. There are no previous studies supported by high certainty evidence as determined by the GRADE assessment [3].

Retention has varied widely in previous follow-up studies of children or adolescents born very preterm [24–30]. The reasons for discontinuation have been many and inconsistent depending on the study settings and follow-up protocols. To our knowledge, there are no previous studies evaluating differences in retention rates between adolescents and adults. Regular contact with study participants and feedback have been suggested to increase retention in follow-up studies of children and adolescents [31]. The PIPARI Study is a unique follow-up study of very preterm infants because of the long follow-up time and high retention (93% at 2 years of corrected age, 84% at 5 years of chronological age and 81% at the age of 11 years) [32]. This might be due to regular contacts with families due to the study protocol and providing the feed-back of the results. The effect of pre-notifications might be even more remarkable in follow-up studies with lower retention.

A major strength of the present study was that the CONSORT guidelines were followed accurately [16]. The study coordinator precisely coordinated the sending of pre- and post-notifications and recording the returned approval forms and questionnaires. A possible limitation was that the number of participants was relatively small in each group. The observed differences between the groups might have become more distinct, if the number of participants had been higher. The neonatal background characteristics were compared between adolescents born very preterm who received pre-notifications and those who did not without any difference. We lacked the equivalent information regarding controls born full term. Our study cohort included more controls than adolescents born very preterm, which enables generalization of the results on study populations including adolescents born full term. In this study, there were participants in both groups, who returned the approval forms, but not the questionnaires, despite the post-notifications. To prevent this phenomenon, further research is needed.

## Conclusion

Our study expands the knowledge of the impact of pre-notifications on the return rates of approval forms and questionnaires in adolescents born very preterm and full term. We showed that the pre-notification letters decreased the need for post-notifications and may increase retention. Based on our findings, sending pre-notification letters are recommended in future follow-up studies.

## Supplementary Information

**Additional file 1.** Abbreviations with references of the Figure 1.

**Additional file 2.** Figures of the pre-notification letters to adolescents born very preterm and control group.

## Data Availability

The datasets used and analyzed during the current study are available from the corresponding author on reasonable request.

## Abbreviations

GRADE: Grading of Recommendations, Assessment, Development and Evaluations
MRI: Magnetic resonance imaging
SMS: Short message service
SWAT: Study within a trial

## References

[1] Schulz KF, Grimes DA (2002). Sample size slippages in randomised trials: exclusions and the lost and wayward. Lancet.

[2] Fewtrell MS, Kennedy K, Singhal A, Martin RM, Ness A, Hadders-Algra M (2008). How much loss to follow-up is acceptable in long-term randomised trials and prospective studies?. Arch Dis Child.

[3] Walters SJ, Bonacho Dos Anjos Henriques-Cadby I, Bortolami O, Flight L, Hind D, Jacques RM (2017). Recruitment and retention of participants in randomised controlled trials: a review of trials funded and published by the United Kingdom Health Technology Assessment Programme. BMJ Open.

[4] Treweek S, Bevan S, Bower P, Campbell M, Christie J, Clarke M (2018). Trial Forge Guidance 1: what is a Study Within A Trial (SWAT)?. Trials..

[5] Gillies K, Kearney A, Keenan C, Treweek S, Hudson J, Brueton VC, et al. Strategies to improve retention in randomised trials. Cochrane Database Syst Rev. 2021;2021:1465–1858. 10.1002/14651858.MR000032.pub3 PMC8092429 33675536

[6] Ashby R, Turner G, Cross B, Mitchell N, Torgerson D (2011). A randomized trial of electronic reminders showed a reduction in the time to respond to postal questionnaires. J Clin Epidemiol..

[7] Starr K, McPherson G, Forrest M, Cotton SC (2015). SMS text pre-notification and delivery of reminder e-mails to increase response rates to postal questionnaires in the SUSPEND trial: a factorial design, randomised controlled trial. Trials..

[8] Severi E, Free C, Knight R, Robertson S, Edwards P, Hoile E (2011). Two controlled trials to increase participant retention in a randomized controlled trial of mobile phone-based smoking cessation support in the United Kingdom. Clinical Trials.

[9] Bradshaw LE, Montgomery AA, Williams HC, Chalmers JR, Haines RH (2020). Two-by-two factorial randomised study within a trial (SWAT) to evaluate strategies for follow-up in a randomised prevention trial. Trials.

[10] Bauer J, Rezaishiraz H, Head K, Cowell J, Bepler G, Aiken M (2004). Obtaining DNA from a geographically dispersed cohort of current and former smokers: use of mail-based mouthwash collection and monetary incentives. Nicotine Tob Res.

[11] Rodgers S, Sbizzera I, Cockayne S, Fairhurst C, Lamb SE, Vernon W (2019). A study update newsletter or Post-it® note did not increase postal questionnaire response rates in a falls prevention trial: an embedded randomised factorial trial. F1000Res..

[12] Goulao B, Duncan A, Floate R, Clarkson J, Ramsay C (2020). Three behavior change theory–informed randomized studies within a trial to improve response rates to trial postal questionnaires. J Clin Epidemiol.

[13] Brozek JL, Canelo-Aybar C, Akl EA, Bowen JM, Bucher J, Chiu WA (2021). GRADE Guidelines 30: the GRADE approach to assessing the certainty of modeled evidence-an overview in the context of health decision-making. J Clin Epidemiol.

[14] Setänen S, Lehtonen L, Parkkola R, Matomäki J, Haataja L (2016). The motor profile of preterm infants at 11 y of age. Pediatr Res.

[15] Harris PA, Taylor R, Minor BL, Elliott V, Fernandez M, O’Neal L (2019). The REDCap consortium: building an international community of software platform partners. J Biomed Inform.

[16] Schulz KF, Altman DG, Moher D (2010). CONSORT 2010 statement: updated guidelines for reporting parallel group randomised trials. BMJ..

[17] Aylward GP, Hatcher RP, Stripp B, Gustafson NF, Leavitt LA (1985). Who goes and who stays: subject loss in a multicenter, longitudinal follow-up study. J Dev Behav Pediatr.

[18] Zook PM, Jordan C, Adams B, Visness CM, Walter M, Pollenz K (2010). Retention strategies and predictors of attrition in an urban pediatric asthma study. Clinical Trials.

[19] Setänen S, Haataja L, Parkkola R, Lind A, Lehtonen L (2013). Predictive value of neonatal brain MRI on the neurodevelopmental outcome of preterm infants by 5 years of age. Acta paediatrica.

[20] Aitken L, Gallagher R, Madronio C (2003). Principles of recruitment and retention in clinical trials. Int J Nurs Pract.

[21] Keding A, Brabyn S, MacPherson H, Richmond SJ, Torgerson DJ (2016). Text message reminders to improve questionnaire response rates. J Clin Epidemiol.

[22] Man M-S, Tilbrook HE, Jayakody S, Hewitt CE, Cox H, Cross B (2011). Electronic reminders did not improve postal questionnaire response rates or response times: a randomized controlled trial. J Clin Epidemiol.

[23] Partha Sarathy P, Kottam L, Parker A, Brealey S, Coleman E, Keding A (2020). Timing of electronic reminders did not improve trial participant questionnaire response: a randomized trial and meta-analyses. J Clin Epidemiol.

[24] Drysdale SB, Lo J, Prendergast M, Alcazar M, Wilson T, Zuckerman M (2014). Lung function of preterm infants before and after viral infections. Eur J Pediatr.

[25] Johnson AH, Peacock JL, Greenough A, Marlow N, Limb ES, Marston L (2002). High-frequency oscillatory ventilation for the prevention of chronic lung disease of prematurity. N Engl J Med.

[26] Ambalavanan N, Tyson JE, Kennedy KA, Hansen NI, Vohr BR, Wright LL (2005). Vitamin A supplementation for extremely low birth weight infants: outcome at 18 to 22 months. Pediatrics.

[27] Zivanovic S, Peacock J, Alcazar-Paris M, Lo JW, Lunt A, Marlow N (2014). Late outcomes of a randomized trial of high-frequency oscillation in neonates. N Engl J Med.

[28] Larroque B, Ancel P-Y, Marchand-Martin L, Cambonie G, Fresson J, Pierrat V (2011). Special care and school difficulties in 8-year-old very preterm children: the Epipage cohort study. PLoS One.

[29] O’Reilly H, Ni Y, Johnson S, Wolke D, Marlow N (2021). Extremely preterm birth and autistic traits in young adulthood: the EPICure study. Mol Autism.

[30] Roberts G, Burnett AC, Lee KJ, Cheong J, Wood SJ, Anderson PJ (2013). Quality of life at age 18 years after extremely preterm birth in the post-surfactant era. J Pediatr.

[31] MacBean V, Drysdale SB, Zivanovic S, Peacock JL, Greenough A (2019). Participant retention in follow-up studies of prematurely born children. BMC Public Health.

[32] Setänen S. Prediction of neurodevelopment and neuromotor trajectories in very preterm born children up to 11 years of age: PIPARI study. Turku: University of Turku; 2016.

