# Supplementary material for: Pre-notifications increase retention in a 17-year follow-up of adolescents born very preterm
Source: Trials. 2023 Jul 26;24:477. doi: 10.1186/s13063-023-07390-1 (PMC10373294; doi:10.1186/s13063-023-07390-1)
Supplement: Supplementary file 1 — Additional file 1. Abbreviations with references of the Figure 1. [file 13063_2023_7390_MOESM1_ESM.docx]

Additional File 1.

Abbreviations with references of the Figure 1.

BAEP = Brainstem auditory evoked potential

Dubowitz = Dubowitz neurologic examination [1]
Baby Day Diary [2]
HINE = Hammersmith Infant Neurological Examination [3]
Bayley-II = Bayley Scales of Infant Development, II [4]
SSQ = Social support questionnaire
EPDS = Edinburgh Postnatal Depression Scale [5]
PC-ERA = Parent-Child Early Relational Assessment [6]
BDI = Beck Depression Inventory [7]
PSI = Parenting Stress Index [8]
SoC-13 = Sence of Coherence Scale [9]
Feeding Q = Feeding-questionnaire
WMCI = Working Model of the Child Interview Coding Manual [10]
Development of early vocalization [11]
MCDI = MacArthur Communicative Development Inventory [12]

Reynell = Reynell Developmental Language Scales III [13]
CBCL = Child Behavior Checklist [14]
WPPSI-R = Wechsler Preschool and Primary Scale of Intelligence [15,16]
NEPSY II = A developmental neuropsychological assessment, 2nd edition [17]

Boston naming test [18]
Academic skills [19]
FAD = McMaster Family Assessment
17D = 17-dimensional questionnaire [20]
Touwen = Touwen neurological examination [21]
Movement ABC-2 = Movement Assessment Battery for Children – 2 [22]
WISC-IV = Wechsler intelligence scale for children [15,16]
BRIEF = Behavior Rating Inventory of Executive Function [23]
DCDQ’07 = Developmental Coordination Disorder Questionnaire 2007 [24]
Viivi (5–15) = Five to Fifteen (FTF) [25]
WMTB-C = Working Memory Test Battery for Children [26]
MASK = Multisource assessment of children’s social competence [27]
PNDL = Peer Network and Dyadic Loneliness Scale [28,29]

1. Dubowitz LMS, Dubowitz V, Mercuri E. The neurological assessment of the preterm and full-term newborn infant. 2nd edition. London; 1999.

2. Barr RG, Kramer MS, Boisjoly C, McVey-White L, Pless IB. Parental diary of infant cry and fuss behaviour. Arch Dis Child. 1988;63:380–7.

3. Haataja L, Mercuri E, Regev R, Cowan F, Rutherford M, Dubowitz V, et al. Optimality score for the neurologic examination of the infant at 12 and 18 months of age. J Pediatr. 1999;135:153–61.

4. Bailey N. Bayley Scales of Infant Development. 2nd Edition. San Antonio: Psychological Corporation; 1993.

5. Cox JL, Holden JM, Sagovsky R. Detection of Postnatal Depression. British Journal of Psychiatry. 1987;150:782–6.

6. Clark R. The Parent-Child Early Relational Assessment: A Factorial Validity Study. Educ Psychol Meas. 1999;59:821–46.

7. Beck AT. An Inventory for Measuring Depression. Arch Gen Psychiatry. 1961;4:561.

8. Abidin RR. Parenting stress index : professional manual. 3rd Edition. Psychological Assessment Resources, Odessa; 1995.

9. Antonovsky A. Unraveling the mystery of health. How people manage stress and stay well. . San Francisco: Jossey-Bass Publishers; 1987.

10. Zeanah CH, Benoit D. Working Model of the Child Interview. New Orleans: Louisiana State University School of Medicine; 1996.

11. Lyytinen P. Ääntelyn ja motoriikan kehityksen seurantamenetelmä. Jyväskylä: Niilo Mäki Instituutti; 2000.

12. Fenson L, Dale PS, Reznick JS, Bates E, Thal DJ, Pethick SJ. Variability in early communicative development. Monogr Soc Res Child Dev. 1994;59:1–173; discussion 174-85.

13. Edwards S. Reynell developmental language scales 3. Berkshire: NFER-Nelson Health & Social Care ; 1997.

14. Achenbach TM. Manual for the Child Behaviour Checklist/1 1⁄2-5 and 2001 profile. Burlington: University of Vermont Department of Child Psychiatry ; 2001.

15. Wechsler intelligence scale for children, IV. Käsikirja I, Esitys- ja pisteitysohjeet (Handbook I. administration and scoring) . Jyväskylä psykologien kustannus 2011; 2011.

16. Wechsler intelligence scale for children, IV. Käsikirja II. Teoriatausta, Standardointi ja Tulkinta (Handbook II. theoretical background, standardization and interpretation). Jyväskylä Psykologien Kustannus ; 2011.

17. Korkman M, Kirk U, Kemp S. A developmental neuropsychological assessment, 2nd version. Test materials and manual. . San Antonio: Harcourt Assessments ; 2007.

18. Kaplan E. Boston naming test. Philadelphia: Lea & Febiger; 1983.

19. Alanko O, Niemi P, Munck P, Matomäki J, Turunen T, Nurmi J-E, et al. Reading and math abilities of Finnish school beginners born very preterm or with very low birth weight. Learn Individ Differ. 2017;54:173–83.

20. Apajasalo M, Rautonen J, Holmberg C, Sinkkonen J, Aalberg V, Pihko H, et al. Quality of life in pre-adolescence: A 17-dimensional health-related measure (17D). Quality of Life Research. 1996;5:532–8.

21. Hadders-Algra M. Neurological examination of the child with minor neurological dysfunction. London: Mac Keith Press; 2010.

22. Henderson SE, Sugdan DA, Barnett A. Movement Assessment Battery for Children (examiner’s manual). London: Pearson Assessment; 2007.

23. Gioia GA, Isquith PK, Guy SC, Kenworthy L. Behavior Rating Inventory of Executive Function. Child Neuropsychology. 2000;6:235–8.

24. Wilson BN, Crawford SG, Green D, Roberts G, Aylott A, Kaplan BJ. Psychometric Properties of the Revised Developmental Coordination Disorder Questionnaire. Phys Occup Ther Pediatr. 2009;29:182–202.

25. Kadesjö B, Janols L-O, Korkman M, Mickelsson K, Strand G, Trillingsgaard A, et al. The FTF (Five to Fifteen): the development of a parent questionnaire for the assessment of ADHD and comorbid conditions. Eur Child Adolesc Psychiatry. 2004;13:iii3–13.

26. Pickering S, Gathercole S. Working me- mory test battery for children (WMTB-C). Manual. . London: Pearson Educational Ltd ; 2001.

27. Junttila N, Voeten M, Kaukiainen A, Vauras M. Multisource assessment of children’s social competence. Educ Psychol Meas. 2006;66:874–95.

28. Junttila N, Vauras M. Loneliness among school-aged children and their parents. Scand J Psychol. 2009;50:211–9.

29. Hoza B, Bukowski WM, Beery S. Assessing Peer Network and Dyadic Loneliness. J Clin Child Psychol. 2000;29:119–28.
