# Supplementary figures and images for: Pre-notifications increase retention in a 17-year follow-up of adolescents born very preterm
Source: Trials. 2023 Jul 26;24:477. doi: 10.1186/s13063-023-07390-1 (PMC10373294; doi:10.1186/s13063-023-07390-1)

Additional File 2.

The pre-notification letter to adolescent born very preterm and controls.


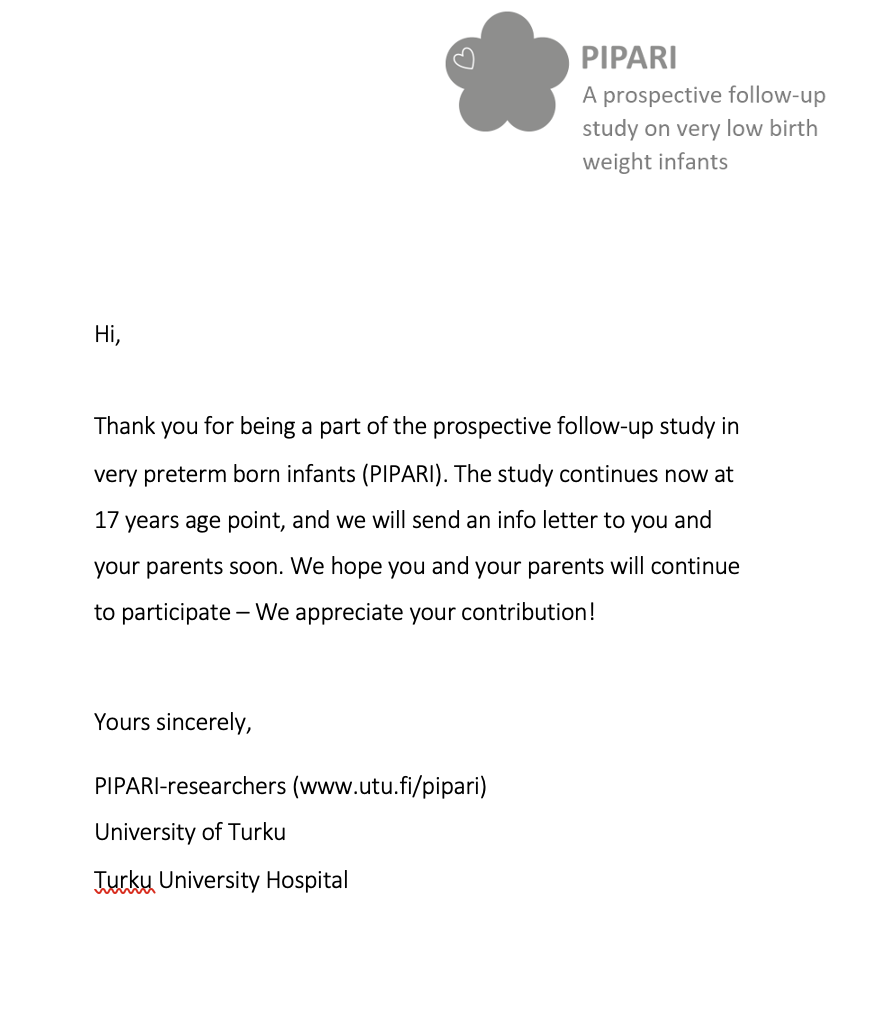


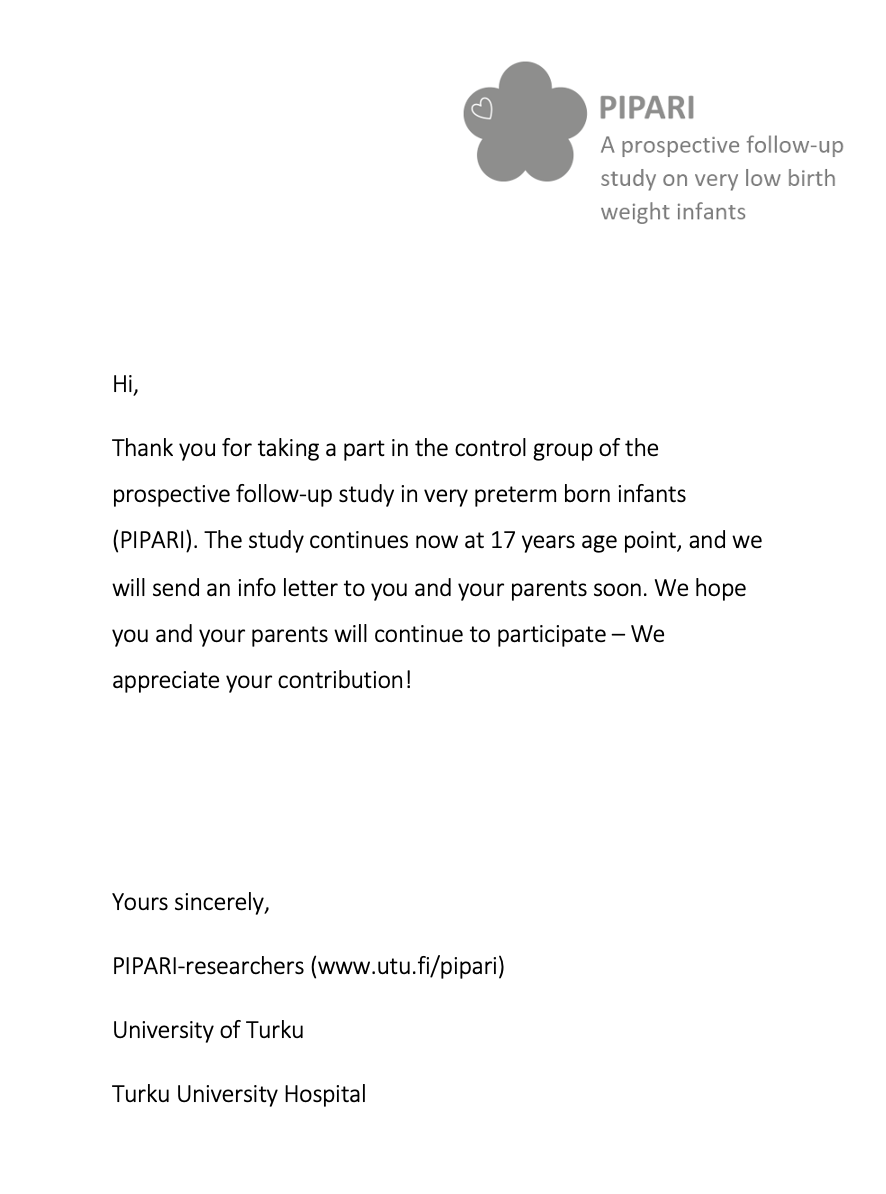

Supplement: Supplementary file 2 — Additional file 2. Figures of the pre-notification letters to adolescents born very preterm and control group. [file 13063_2023_7390_MOESM2_ESM.docx]
